# Physiological swelling imaging in human calf under stocking compression by sparse Bayesian learning implemented into electrical impedance tomography (SBL-EIT)

**DOI:** 10.1007/s13534-025-00524-1

**Published:** 2025-11-22

**Authors:** Kota Asano, Ryoma Ogawa, Prima Asmara Sejati, Shinsuke Akita, Masahiro Takei

**Affiliations:** 1 Department of Mechanical Engineering, Graduate School of Science and Engineering, Chiba University, 1-33, Yayoicho, Inage-ku, Chiba-shi, Chiba, 263-8522 Japan; 2 Department of Plastic, Reconstructive and Aesthetic Surgery, Chiba University Hospital, 1-8-1, Inohana, Chuo-ku, Chiba-shi, Chiba, 260-8677 Japan; 3 Department of Electrical Engineering and Informatics, Vocational College, Universitas Gadjah Mada, Yogyakarta, 55281 Indonesia

**Keywords:** Physiological swelling, Stocking compression, Sparse Bayesian learning, Electrical impedance tomography

## Abstract

Physiological swelling in human calf has been imaged under stocking compression by the sparse Bayesian learning implemented into electrical impedance tomography (SBL-EIT) to evaluate the in situ treatment effect of various compression pressures. SBL-EIT reconstructs conductivity distribution $\Delta {\boldsymbol{\upsigma}}$ to image excessive extracellular fluid in subcutaneous adipose tissue (SAT), indicating the susceptibilities to physiological swelling due to various compression pressures. The SBL-EIT was applied to the imaging of eight-subject calves during prolonged standing under three types of net compression pressures *P*^net^ measured by pressure sensor – strong pressure: *P*^net, Strong^ = 11.9 $\pm$ 2.0 mmHg, weak pressure: *P*^net, Weak^ = 4.47 $\pm$ 3.1 mmHg, and control pressure: *P*^net, Control^ = 0.00 $\pm$ 0.0 mmHg, respectively. From the experimental results, the spatial-mean conductivity ⟨**σ**⟩^α2^ with two pre-processing steps to eliminate undesirable effects, i.e., the difference in skin condition and effect of wearing stockings itself, is the highest in the case of stocking with control pressure, followed by weak and strong pressures across all subjects. Moreover, the ⟨**σ**⟩^α*2*^ has a strong positive correlation with the conventional inversed impedance 1/*z*^BIA^ by bioelectrical impedance analysis (BIA) (a correlation coefficient 0.528 < *R* < 0.990; *n* = 19 and *p* < 0.05), which is mainly increased during the prolonged standing. Moreover, various susceptibilities to physiological swelling are investigated based on the increase in $\Delta {\boldsymbol{\upsigma}}$ for each subject, which is associated with subject external factors such as postural changes and circumference and internal factors like SAT.

## Introduction

Physiological swelling is often developed by circulation-related diseases such as lymphedema [1], chronic venous insufficiency [2], and chronic kidney disease [3], which results in the accumulation of excessive extracellular fluid in human calf. Continuous daily treatment is essential since physiological swelling deteriorates the quality of life (QOL), such as immobility, discomfort, and pain [4]. For treating physiological swelling, stocking compression is a promising method of improving blood flow by applying high pressure around the calf to assist the muscle pump without disturbing daily activities [5]. Since the pressure of stocking compression strongly depends on subject-specific physiological factors such as calf circumference and subcutaneous adipose tissue (SAT) thickness, the objective evaluation of the treatment effect across a broad area under the in situ treatment is highly demanded.

The effect of stocking compression in physiological swelling is conventionally evaluated by circumferential and volumetric measurements with a measuring tape because of its simplicity [6]. For example, calf circumference is decreased by 1.0% after 30 min of stocking compression with a pressure gradient of 15.8 to 26.3 mmHg, while calf volume is reduced by up to 50% after 7 h of stocking compression [7]. However, the circumferential and volumetric measurements are insufficient because physiological expansion occurs primarily in SAT, which limits the sensitivity of the simple external measurement.

In order to achieve the objective evaluation, magnetic resonance imaging (MRI) [8] and X-ray computed tomography [9] are typically used to measure leg volume, which might be applied before and after the stocking compression. Nevertheless, these modalities are not practical for daily usage of stocking compression because of high cost, bulkiness, and lack of convenience and rapid assessment; moreover, the evaluation under the in situ treatment is impossible. As an alternative modality, bioelectrical impedance analysis (BIA) is paid attention due to its non-invasive and simple measurement [10] and is widely applied to physiological swelling evaluation [11]. However, the objectivity of BIA for evaluating treatment effects remains limited, as it provides only whole-limb estimates and cannot monitor the local changes occurring specifically under stocking compression, which are spatially and temporally heterogeneous.

In order to address the problem mentioned above, electrical impedance tomography (EIT) is more desirable than the conventional evaluation because EIT images are “spatial” and “temporal” changes in the extracellular fluid by a simple, non-invasive, harmless, low-cost, and real-time [12]. Generally, EIT images conductivity distribution to evaluate body fluids such as urine [13], lung edema [14], and brain hemorrhage [15]. Under the circumstances, spatiotemporal change due to physiological swelling during prolonged standing and post-exercise was already imaged by EIT with a wearable sensor [12]. To the best of our knowledge, this study is the first to enable in situ monitoring of physiological swelling inside the treatment area while wearing compression stockings using EIT. Moreover, imaging accuracy, particularly for SAT, was further improved by introducing Sparse Bayesian learning (SBL-EIT), allowing more precise localization of spatio-temporal changes [16]. Therefore, we applied SBL-EIT to image physiological swelling in the human calf under stocking compression, mainly focusing on SAT, in order to leverage its improved spatial resolution for more detailed evaluation.

The objectives of this study are (1) to apply SBL-EIT to physiological swelling imaging under various stocking compressions while considering the difference in skin conditions and (2) to investigate whether EIT can detect changes in leg swelling under various compression pressures by comparing with conventional methods.

## Experiments and evaluation

### Stocking and EIT sensor

**Fig. 1:**
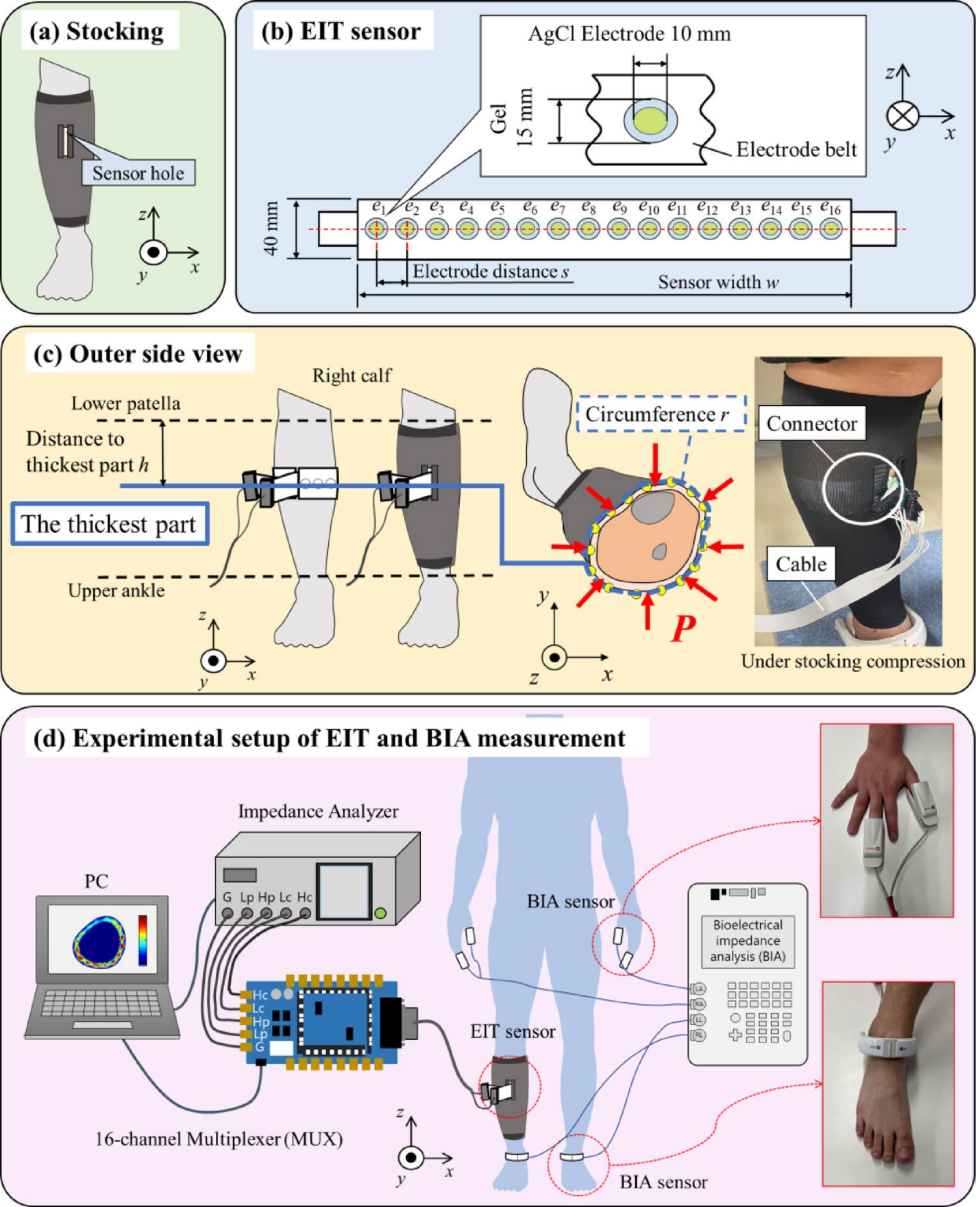
**a** Stocking with a sensor hole, **b** EIT sensor, **c** outer side view and **d** experimental setup of EIT and BIA measurements

Figure 1a shows the schematic of a stocking with a sensor hole, Fig. 1b shows an inner front view of the EIT sensor, and Fig. 1c shows an outer side view of the EIT sensor covered by the stocking. Three types of stockings which cover the ankle to the calf with various compression pressures are prepared: strong pressure (Gracewell®, Japan), weak pressure (MediQtto®, Japan), and control pressure (Caseeto, Japan). The stockings with strong and weak pressures compress the calf from the distal part to the proximal part with the pressure gradient while the stocking with control pressure does not compress the calf. The holes are equally perforated in all stockings for EIT sensor connection, and reinforcement with hard plastics is used to prevent the stockings from tearing through the holes.

In order to measure the net compression pressure, the contact pressure during the stocking compression at the height of the EIT sensor hole is measured by a pressure sensor comprised of two piezoresistive sensors (MF02A-N-221-A01, Taiwan alpha electronic CO., LTD.). Calibration was performed to estimate pressure from resistance values based on the relationship between sensor resistance, mass, and the base area of various weights. The sensor was then positioned between the stocking and the calf through the sensor hole shown in Fig. 1c, and the net compression pressure *P*^net^ was calculated using the above calibration. As a result, three types of stockings are defined as strong, weak, and control pressures according to *P*^net^. In order to ensure standardized experimental conditions, a single stocking size was selected for each type to provide a clear understanding of the relationship between calf circumference and *P*^net^.

In this study, the EIT sensor consists of the 16 AgCl electrodes marked as ${e}_{1}$, ${e}_{2}$, ${e}_{{\ell}}$, …, ${e}_{L}$ (*L* = 16) with an electrode radius of 10 mm and gel radius of 15 mm which are equidistantly embedded inside the white polyethylene terephthalate substrate. Since the substrate is non-stretchable, four sizes of EIT sensors (S, M, L, LL) with electrode distance *s* and sensor length *w* are prepared according to the subject's calf circumference, as shown in Fig. 1b and Table 1. Furthermore, the EIT sensor is attached to the thickest part of the right calf, and the stocking is covered over the EIT sensor, as shown in Fig. 1c.

**Table 1:** Circumference of the thickest part *r*, distance from lower patella to thickest part *h*, sensor size for each subject, and sensor width *w*, electrode distance *s* for each sensor size

Subject	*r*[mm]	*h*[mm]	Size	*w*[mm]	*s*[mm]
*S*_1_	335	95	S	330	20
*S*_2_	339	112			
*S*_3_	340	140			
*S*_4_	355	140	M	360	22
*S*_5_	370	145			
*S*_6_	370	165			
*S*_7_	400	135	L	390	24
*S*_8_	420	150	LL	420	26

### Experimental setup

Figure 1d shows the experimental setup of EIT and BIA measurements. The experimental setup of EIT consists of five units: an EIT sensor attached around the right calf, a stocking covering over the EIT sensor, an impedance analyzer (IM3570, HIOKI, Japan), a 16-channel multiplexer (made by Takei Lab), and a personal computer (PC) [17]. The impedance analyzer measures the impedance **Z** with a specific frequency *f* controlled by the PC through a USB connection to perform the image reconstruction. The multiplexer is built based on an embedded advanced RISC machine (ARM) Cortex-M4 core microcontroller developed in our previous study [18]. The multiplexer is employed to select the route between the input and output signal from the impedance analyzer on each ${e}_{{\ell}}$ in the EIT sensor. The connection between the multiplexer and impedance analyzer is divided into 4 ports: ${H}_{c}$, ${H}_{p}$, ${L}_{c}$, and ${L}_{p}$. The BIA measurement setup comprises a BIA system and BIA electrodes (two for hands and two for feet) (InBody S10, InBody Japan Inc.), which measures the conventional impedance *z*^BIA^ as a segmental value for the right leg (RL). According to our previous studies, EIT and BIA measurements do not interfere with each other even if the electrodes are still attached unless both measurements are performed simultaneously [19].

### Experimental protocol and methods

Before conducting the experiment, net compression pressure *P*^net^ is measured by the pressure sensor in the case of three types of stockings for subjects *S*_1_–*S*_8_. Table 2 shows the net compression pressure *P*^net^ results with the three types of stockings for each subject.

**Table 2:** Net compression pressure *P*^net^ with the three types of stockings for subjects *S*_1_ – *S*_8_

Pressure [mmHg]	*S*_1_	*S*_2_	*S*_3_	*S*_4_	*S*_5_	*S*_6_	*S*_7_	*S*_8_
Strong	9.41	12.8	12.5	10.1	10.8	9.93	14.8	14.5
Weak	1.12	3.30	6.42	5.35	2.26	1.08	5.21	11.0
Control	0.00	0.00	0.00	0.00	0.00	0.00	0.00	0.00

**Fig. 2:**
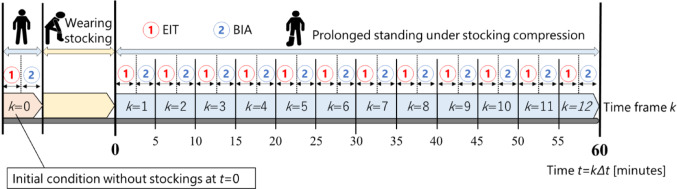
Experiment protocol of physiological swelling imaging in human calf under stocking compression

Figure 2 shows the experimental protocol of physiological swelling imaging in human calf under stocking compression. The experiments were conducted on three days under the same conditions except for the stocking compression. At the beginning of each day, the EIT sensor is attached to the subject’s calf. The stockings with strong, weak, and control pressures are attached to the EIT sensor on day 1, day 2, and day 3, respectively, in order to apply various compression pressures around the calf. Both EIT and BIA measurements are conducted before wearing stockings as an initial condition without stocking compression, at which the time frame is defined as* k* = 0. After wearing stockings, EIT measurement is performed for 1 min, followed by a BIA measurement for 2 min and a 2-min break, resulting in a total cycle duration of ∆*t* = 5 min. In order to induce physiological swelling, the subjects remain standing under stocking compression without minimal movement, repeating 12 times (*k* = 12) until *t* = 55 min from* t* = *k*∆*t*.

The experiment was conducted on four healthy females and four healthy males (*S*_1_–*S*_8_; age 25.1 ± 4.3 years, BMI 22.1 ± 2.8 kg/m^2^, with no history of severe illness or comorbidity) in accordance with the ethical guidelines of the Bioethics Committee in the Faculty of Engineering, Chiba University, Japan, with informed consent. The data acquisition system of EIT is composed of four ports: ${H}_{C}$ (high current), ${H}_{P}$ (high potential), ${L}_{C}$ (low current), and ${L}_{P}$ (low potential) to inject the alternating current ${I}_{{\ell}}$ and measure **Z** on specific electrode ${e}_{{\ell}}$. ${I}_{{\ell}}$ produces an electric potential distribution $\varphi$ that depends on $\sigma$. In this study, an adjacent pattern is utilized due to the advantage of the high sensitivity to the periphery [12] in which the ports ${H}_{C}-{L}_{C}$ and ${H}_{P}-{L}_{P}$ are adjacent. The **Z** is measured at various time frames (*k* = 0–12) with a small constant alternating current ${I}_{{\ell}}$ = 0.5 mA with the frequency *f* = 6.3 kHz considering the current paths that penetrate only the extracellular spaces. The comparison parameter is *z*^*BIA*^ at frequency *f*^BIA^ = 5 kHz in the whole right leg.

### Sparse Bayesian learning implemented into electrical impedance tomography (SBL-EIT)

**Fig. 3:**
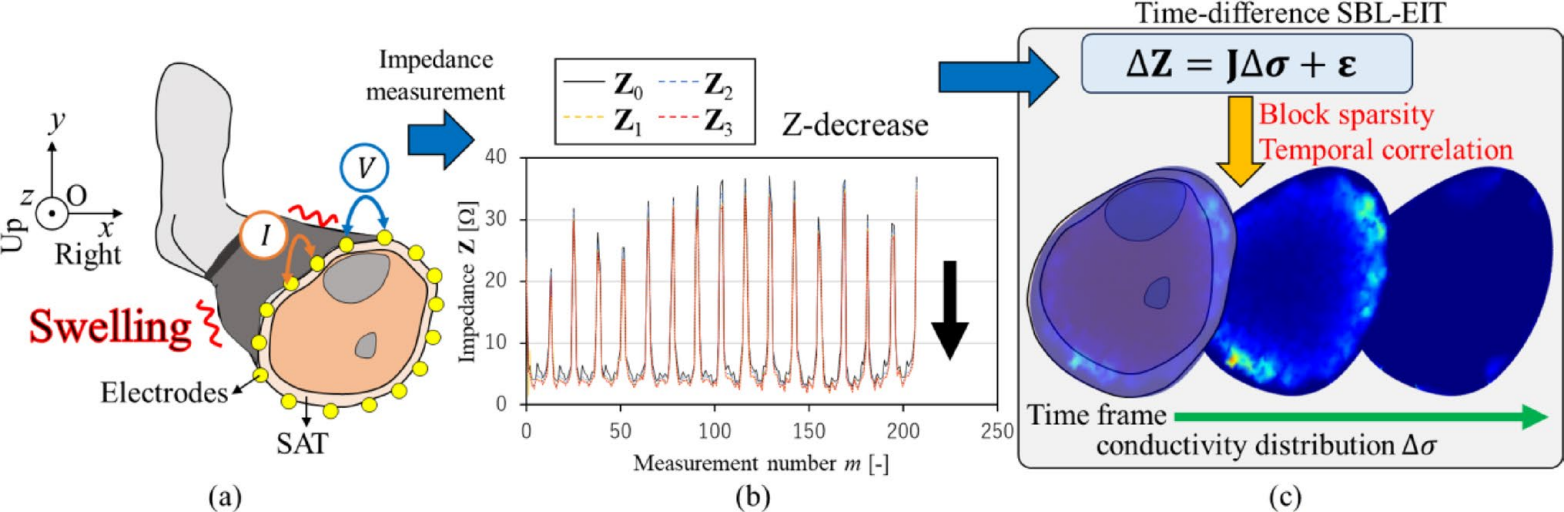
The overview of SBL-EIT. **a** Electrode position in human calf, **b** impedance measurement in various time frames k, and **c** time-difference SBL-EIT algorithm

Figure 3 shows the overview of SBL-EIT with (a) electrode position in human calf, (b) impedance measurement in various time frames *k*, and (c) time-difference SBL-EIT algorithm. Since the mathematical formulation of SBL-EIT is similar to our previous study [17], only the critical information is provided. In EIT, the estimation of the conductivity distribution from surface voltage measurements is formulated as an inverse problem, which is inherently ill-posed and highly sensitive to noise and modeling errors [20]. To stabilize the reconstruction, various regularization approaches have been proposed. In this study, the inverse problem was addressed by a sparse Bayesian learning (SBL) framework, which introduces probabilistic regularization based on sparsity and temporal correlation to enhance the spatial resolution and robustness of the conductivity estimation [16]. As shown in Fig. 3a and b, time-difference impedance Δ**Z**_*k*_ measured at various time frames *k* by an adjacent pattern is incorporated into SBL-EIT. As shown in Fig. 3c, the key assumption for time-difference SBL-EIT is that the sparsity profile of physiological swelling is “slowly time-varying”; therefore, the temporal correlation is desired to be extracted through the iterative reconstruction algorithm. Firstly, Δ**Z**_*k*_ ∈ ℝ^*M*^ is formulated considering both time-dependency and frequency-dependency, which is expressed by.

1 $$\Delta {\mathbf{Z}}_{k}={\mathbf{Z}}_{k,t}-{\mathbf{Z}}_{k,t1}=\mathbf{J}\Delta {{\boldsymbol{\upsigma}}}_{k}+{{\boldsymbol{\upvarepsilon}}}_{k}$$

where *M* is the total number of measurements (*M* = 208), *N* is the total number of elements (*N* = 13), **J** ∈ ℝ^*M*×*N*^ is the sensitivity matrix, Δ**σ**_*k*_ ∈ ℝ^*N*^ is the conductivity distribution at time frame *k* (*k* = 1, 2,*…*, *K*), **ε**_*k*_ = [*ε*_*k*,1_,*…*, *ε*_*k*,*m*_, *…*, *ε*_*k*,*M*_]^*T*^ ∈ ℝ^*M*^ is the noise vector, and superscript *T* is the transpose of a matrix. In order to extract the temporal correlation, the multiple measurement vector is considered and then column-vectorization is applied in Eq. (1), i.e., Δ**Z** = [Δ**Z**_1_,*…*, Δ**Z**_*k*_,*…*, Δ**Z**_*K*_] ∈ ℝ^*M*×*K*^, Δ**σ** = [Δ**σ**_1_,…, Δ**σ**_*k*_,…, Δ**σ**_*K*_] ∈ ℝ^*N*×*K*^, and **ε** = [**ε**_1_,*…*, **ε**_*k*_, *…*, **ε**_*K*_] ∈ ℝ^*M*×*K*^ with time-constant **J** as follows,

2 $$\Delta {\mathbf{Z}}^{\boldsymbol{*}}=\widetilde{\mathbf{J}}\Delta {{\boldsymbol{\upsigma}}}^{\boldsymbol{*}}+{{\boldsymbol{\upvarepsilon}}}^{*}$$

where * is the symbol of column-vectorization, ΔZ* ∈ ℝ^*MK*^, Δσ* ∈ ℝ^*NK*^, ε* ∈ ℝ^*MK*^, $\mathop {\mathbf{J}}\limits^{\sim }$ = **J** ⊗ **I** ∈ ℝ^*MK*×*NK*^, ⊗ is the symbol of the Kronecker product of two matrices, and **I** ∈ ℝ^*K*×*K*^ is the identity matrix.

Secondly, the one-step image reconstruction is performed in a manner of SBL-EIT framework, i.e., the prior probability distribution *p*(Δσ*;** Σ**^*pre*^), the likelihood *p*(ΔZ* |Δσ*; ε*), and the posterior probability distribution *p*(Δσ*|ΔZ*;** Σ**^*post*^) with respect to Δσ* are defined. Here a prior covariance matrix **Σ**^*pre*^ ∈ ℝ^*NK*×*NK*^ consists of a prior variance vector **γ** = [*γ*_1_,…, *γ*_*n*_,…, *γ*_*N*_]^*T*^ ∈ ℝ^*N*^ related to the sparsity, and a positive definite matrix **B** ∈ ℝ^*K*×*K*^ related to the temporal correlation [21],

3 $$p(\Delta{\boldsymbol{\sigma}}*;{{\boldsymbol{\Sigma}}}^{pre})\sim N(0,{{\boldsymbol{\Sigma}}}^{pre})$$

where covariance matrix **Σ**^*pre*^ is as follows,

4 $$ \Sigma ^{{pre}}  = \left[ {\begin{array}{*{20}c}    {\gamma _{1} B} & {} & 0  \\    {} &  \ddots  & {}  \\    0 & {} & {\gamma _{N} B}  \\   \end{array} } \right]  $$

The Δσ* is obtained as the mean vector $\overline{{\Delta {{\boldsymbol{\upsigma}}}*}}$ ∈ ℝ^*NK*^ of multivariate Gaussian distribution with the posterior covariance matrix **Σ**^*post*^ ∈ ℝ^*NK*×*NK*^ which are expressed by

5 $$ \begin{aligned}   \overline{{\Delta \sigma ^{*} }}  &  = \frac{1}{\lambda }\Sigma ^{{post}} \widetilde{{\mathbf{J}}}^{T} \Delta {\mathbf{Z}}^{*}  \\     &  = \left[ {\lambda (\Sigma ^{{pre}} )^{{ - 1}}  + \widetilde{{\mathbf{J}}}^{T} \widetilde{{\mathbf{J}}}} \right]^{{ - 1}} \widetilde{{\mathbf{J}}}^{T} \Delta {\mathbf{Z}}^{*}  \\  \end{aligned}   $$

6 $$\because {{\boldsymbol{\Sigma}}}^{post}={\left[({{\boldsymbol{\Sigma}}}^{pre}{)}^{-1}+\frac{1}{\lambda }{\widetilde{\mathbf{J}}}^{T}\widetilde{\mathbf{J}}\right]}^{-1}$$

where* λ* is the noise variance. The Δ**σ** obtained by Eq. (6) is incorporated into the iteration process with block sparsity, where the hyperparameters *λ*, **γ**, and **B** are learned by means of the expectation–maximization (EM) method [22]. The iteration process is conducted until either the error bound *δ* = ║Δ**σ**^*iter*+1^ − Δ**σ**^*iter*^║_2_/║Δ**σ**^*iter*+1^║_2_ reaches its minimum *δ*^*min*^ or *iter* reaches the maximum iteration number *iter*^*max*^.

The boundary shape in FEM mesh is made based on a typical MRI axial-plane image of human calf [23], which is assumed to be similar for each person. The sensitivity matrix **J** is calculated under the homogeneous condition [12], i.e., **σ** = 1.0 [Sm^−1^]. The minimum error bound and the maximum iteration number for SBL-EIT are set to *δ*^*min*^ = 1 × 10^−2^ and *iter*^*max*^ = 5, respectively, which are optimized by trial and error. The regularization parameter *β* for the temporal correlation is set to *β* = 4 considering noisy **Z**_*k*_ [21].

It must be noted that ${\mathbf{Z}}_{k}$ in the real experiment tends to be excessively noisy due to skin dryness and wearing the stocking itself. Therefore, ${\mathbf{Z}}_{k}$ must be processed by following equations which consider the ideal condition before wearing the stocking, i.e., pre-stocking condition *t*_*k*_ = 0 min:

7 $$ \begin{gathered} {\mathbf{Z}}_{k}^{{\alpha_{1} }} = \alpha_{1} {*}{\mathbf{Z}}_{k} \hfill \\ \because \alpha_{1} = \frac{{{\mathbf{Z}}^{{{\mathrm{Control}}}}_{0} }}{{{\mathbf{Z}}_{0} }} \hfill \\ \end{gathered} $$

where **Z**_*k*_^*α*1^ is calculated from a weight *α*_1_, which considers the noise due to skin condition based on **Z**_*k*_ at *k* = 0 and *k* = 1 by element-wise division. Furthermore, **Z**_*k*_^*α*1^ is further processed by considering the noise due to wearing the stocking to generate **Z**_*k*_^*α*2^ as follows

$$k=0: {\mathbf{Z}}_{k}^{{\alpha }_{2}}={\mathbf{Z}}_{k}^{{\alpha }_{1}}$$

$$k=1-12: {\mathbf{Z}}_{k}^{{\alpha }_{2}}={\alpha }_{2}*{\mathbf{Z}}_{k}^{{\alpha }_{1}}$$

8 $$\because {\alpha }_{2}=\frac{{\mathbf{Z}}_{0}^{{\alpha }_{1}}}{{\mathbf{Z}}_{1}^{{\alpha }_{1}}}$$

where **Z**_*k*_^*α*2^ is calculated from a weight *α*_2_, which considers the noise due to wearing the stocking based on **Z**_*0*_ of three types of stockings. Finally, **Z**_*k*_^*α*2^ is utilized as an input of SBL-EIT reconstruction algorithm. In order to evaluate **Z**_*k*_^*α*2^, the spatial-mean conductivity ⟨${\boldsymbol{\upsigma}}$⟩^*α*2^ and conventional impedance *z*^BIA^ are compared by correlation coefficient *R*.

## Experimental results

### Spatial-mean conductivity

**Fig. 4:**
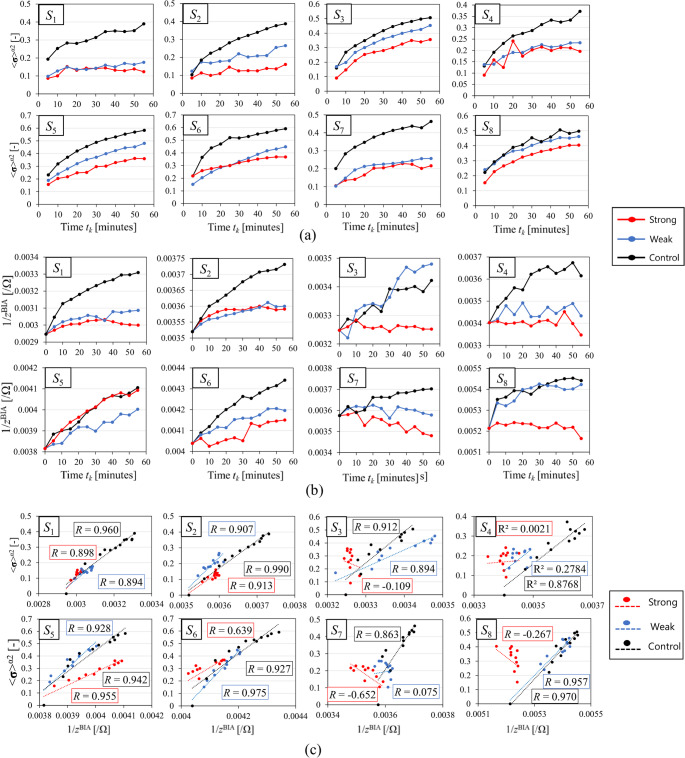
**a** Spatial-mean conductivity ⟨${\boldsymbol{\sigma}}$⟩^*α*2^ processed by considering the pre-stocking condition, **b** Inverse of conventional impedance $1/{z}^{BIA}$ during the prolonged standing for eight subjects (*S*_1_–*S*_8_) in the case of three types of stockings and **c** correlation between ⟨${\boldsymbol{\sigma}}$⟩^*α*2^ and the conventional inversed impedance 1/zBIA for eight subjects in the case of three types of stockings

**Fig. 5:**
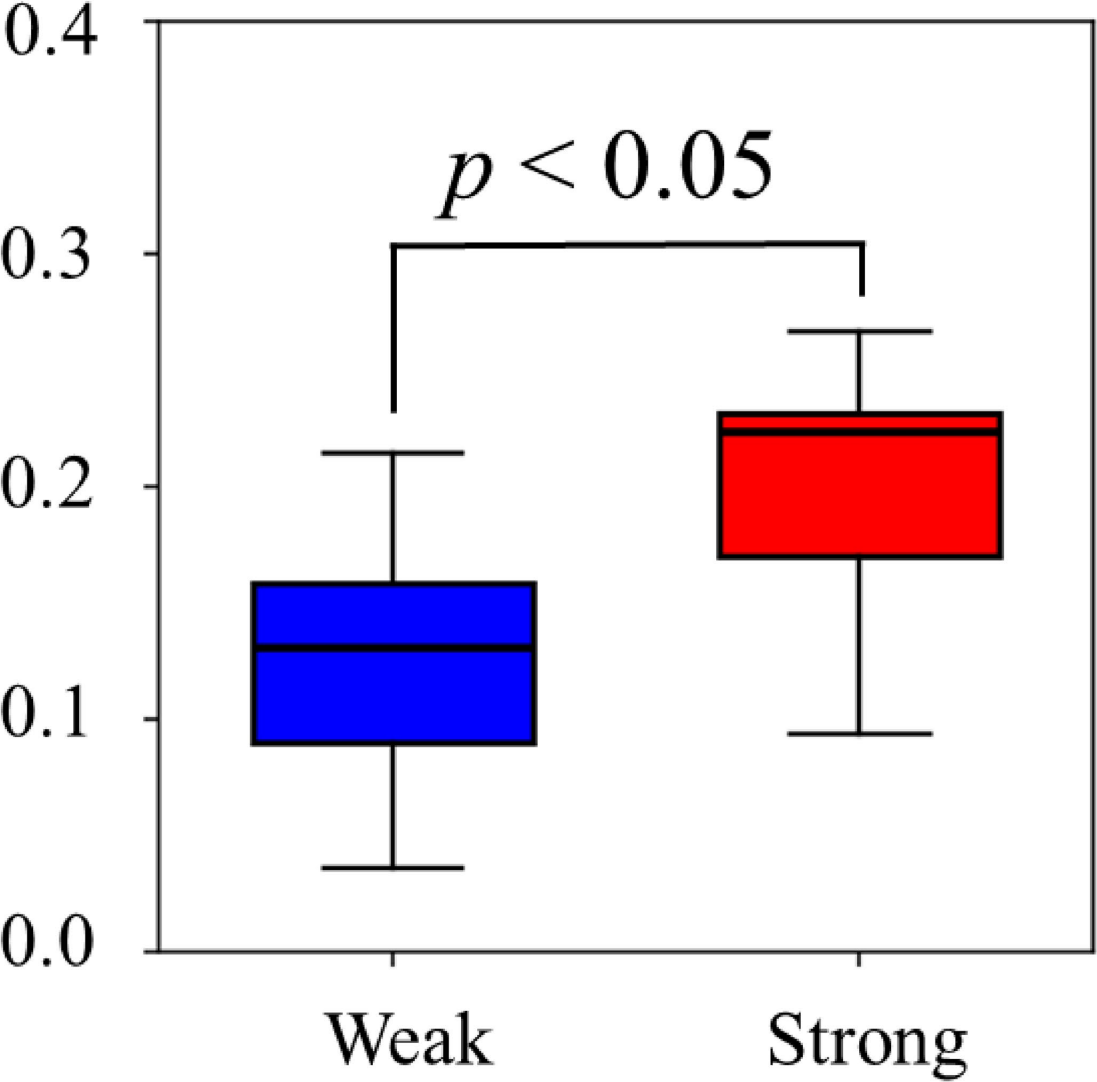
Differences in ⟨$\upsigma$⟩^*α*2^ between stocking with control and strong or weak pressure at *t*_*k*_ = 55 min for eight subjects

Figure 4a shows the spatial-mean conductivity ⟨${\boldsymbol{\upsigma}}$⟩^*α*2^ processed by considering the pre-stocking condition, Fig. 4b shows the conventional inversed impedance 1/*z*^BIA^ during the prolonged standing for eight subjects in the case of three types of stockings. The ⟨${\boldsymbol{\upsigma}}$⟩^*α*2^ is highest in the case of control pressure stocking, followed by stockings with weak and strong pressures across all subjects. The ⟨${\boldsymbol{\upsigma}}$⟩^*α*2^ is mainly increased over time. At *t*_*k*_ = 55 min, the differences in ⟨${\boldsymbol{\upsigma}}$⟩^*α*2^ between the stocking with control pressure and stockings with strong or weak pressure, denoted as ⟨${\boldsymbol{\upsigma}}$⟩^*α*2_Control−Strong^ and ⟨${\boldsymbol{\upsigma}}$⟩^*α*2_Control−Weak^, are statistically significant across all subjects (*p* < 0.05) as shown in Fig. 5, indicating the ability of SBL-EIT to represent the differences in treatment effect of various compression pressures.

Figure 4c shows the correlation between ⟨${\boldsymbol{\upsigma}}$⟩^*α*2^ and the conventional inversed impedance 1/*z*^BIA^ for eight subjects in the case of three types of stockings. The ⟨${\boldsymbol{\upsigma}}$⟩^*α*2^ has a strong positive correlation with the conventional inversed impedance 1/*z*^BIA^ by bioelectrical impedance analysis (BIA) (a correlation coefficient 0.528 < *R* < 0.990; *n* = 19 and *p* < 0.05), which is mainly increased during the prolonged standing. Low or negative correlations are observed in some cases (Strong—*S*_3_: *R* =  − 0.109, *S*_4_: *R* = 0.045, *S*_7_: *R* =  − 0.652, *S*_8_: *R* =  − 0.267, Weak—*S*_7_: *R* = 0.075), which are excluded from the analyzed data. The reasons for these cases are considered to be a) fundamental differences in the methods, which results in different trends in ⟨${\boldsymbol{\upsigma}}$⟩^*α*2^ and the conventional inversed impedance 1/*z*^BIA^ as shown in Fig. 4c, and b) inherent skin dryness, especially at ankle position in BIA measurements, respectively, which is discussed in Sect. 4.3.

### Image reconstruction of conductivity distribution by SBL-EIT

**Fig. 6:**
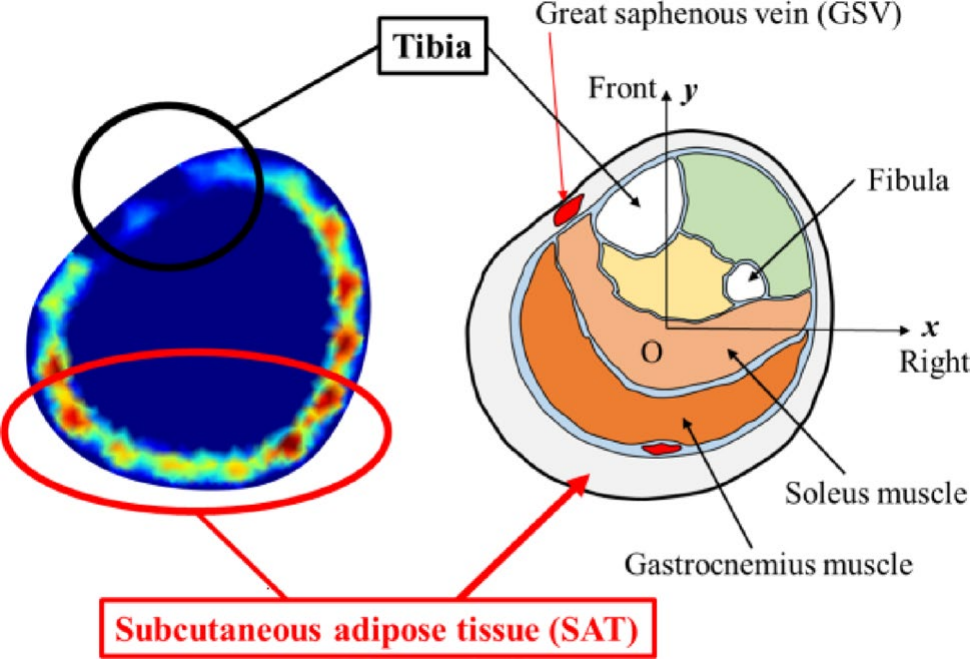
Interpretation of reconstructed images of $\Delta {\boldsymbol{\upsigma}}$ by SBL-EIT

Figure 6 shows the interpretation of reconstructed images of $\Delta {\boldsymbol{\upsigma}}$ by SBL-EIT, using the case of control pressure stockings in *S*_8_ at *t*_*k*_ = 55 min as a representative example. Comparison with the cross-sectional view of the tibia shows that the change in $\Delta {\boldsymbol{\upsigma}}$ occurs mainly in the subcutaneous adipose tissue (SAT) layer. In contrast, the front side of the leg near the tibial shows smaller changes in $\Delta {\boldsymbol{\upsigma}}$.

Table 3 shows the conductivity distribution $\Delta {\boldsymbol{\upsigma}}$ reconstructed by SBL-EIT during the prolonged standing for eight subjects in the case of three types of stockings. Qualitatively, similar to Fig. 6, the changes in Δ ${\boldsymbol{\upsigma}}$ occurred mainly in SAT across all cases where Δ $\upsigma$ is increased over time. Similarly to the ⟨${\boldsymbol{\upsigma}}$⟩^*α2*^, Δ ${\boldsymbol{\upsigma}}$ is highest in the case of control pressure stocking, followed by weak and strong pressure stockings across all subjects. Interestingly, the local conductivity change of ${\Delta {\boldsymbol{\upsigma}}}_{k}$ for each subject is different, which indicates various susceptibilities to Table 3. The various susceptibilities of the relationship between SAT thickness and net compression pressure *P* are discussed in the next section.

**Table 3:**
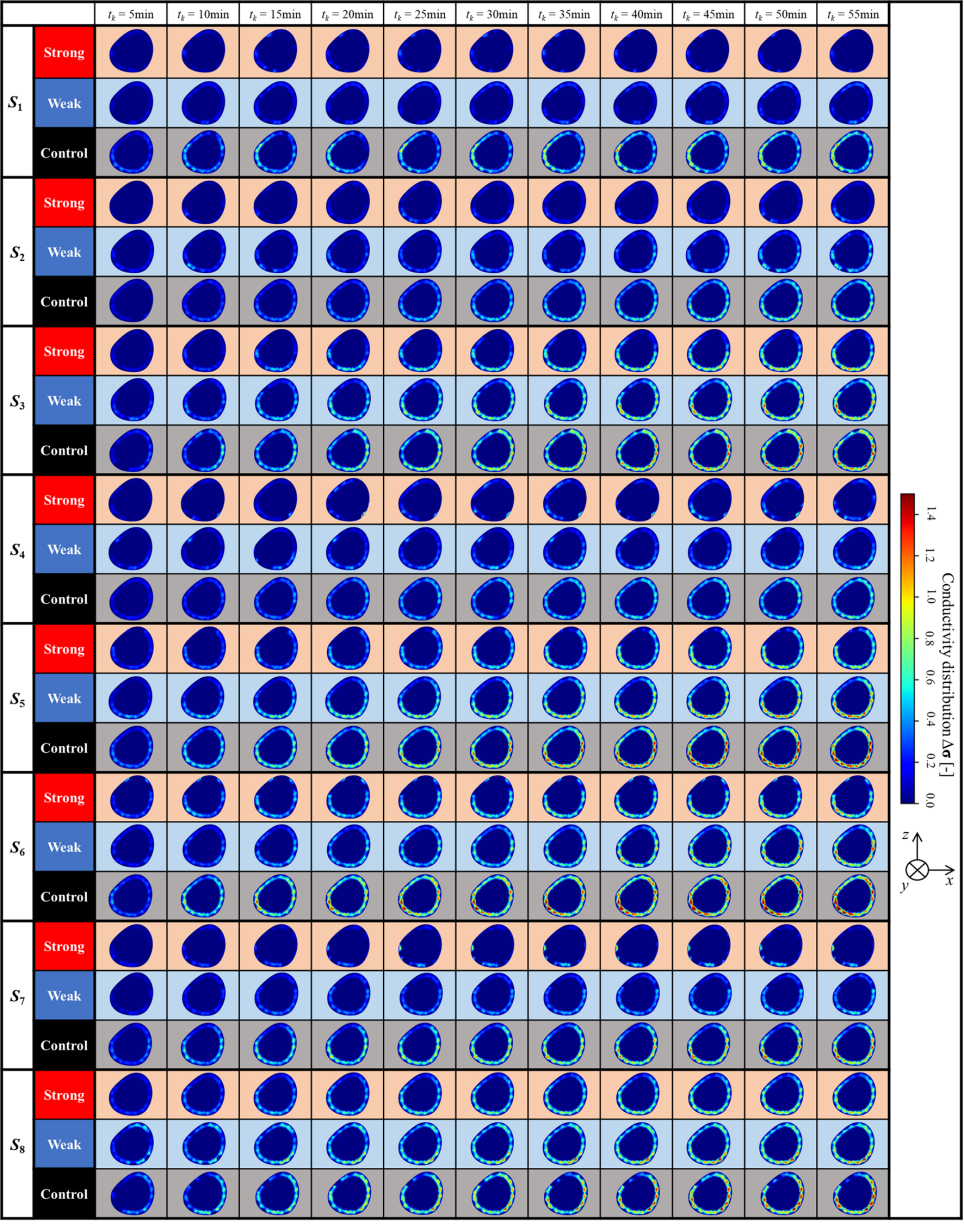
The conductivity distribution Δ ${\boldsymbol{\sigma}}$ reconstructed by SBL-EIT for three subjects in the cace of three types of stockings

## Discussion

### Treatment effect of various compression pressures

**Fig. 7:**
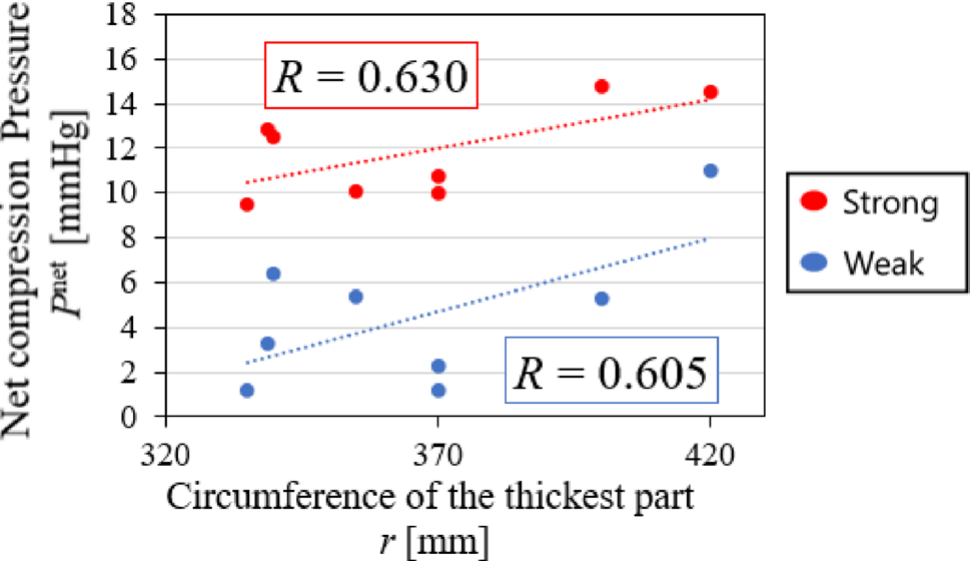
Relationship between circumference of the thickest part *r* and the net compression pressure *P*^net^ of the stockings with Strong and Weak pressure for each subject

The primary objective of this study is to evaluate the sensitivity of EIT to various compression pressures; therefore, all subjects were measured using stockings of identical size to ensure consistency in experimental conditions. However, *P*^net^ varies from subject to subject, even for the same type of stockings, which is associated with the subject’s circumference. Figure 7 shows the relationship between circumference of the thickest part *r* and *P*^net^ of the stockings with Strong and Weak pressure for each subject. Moderate positive correlations (Strong: *R* = 0.630, Weak: *R* = 0.605) between *r* and *P*^net^ are observed, suggesting the importance of selecting appropriate stockings based on circumference. the same stocking is used for all subjects. Furthermore, ⟨${\boldsymbol{\sigma}}$⟩^α2^ in Fig. 4a indicates that treatment effects are observed even with the Weak stocking, suggesting that SBL-EIT may be capable of detecting such subtle changes. Therefore, in clinical practice, objectively evaluating such subtle changes in the in situ treatment effects of various compression pressures is essential, taking into account external factors such as postural changes and circumference.

### Various susceptibilities to physiological swelling

**Fig. 8:**
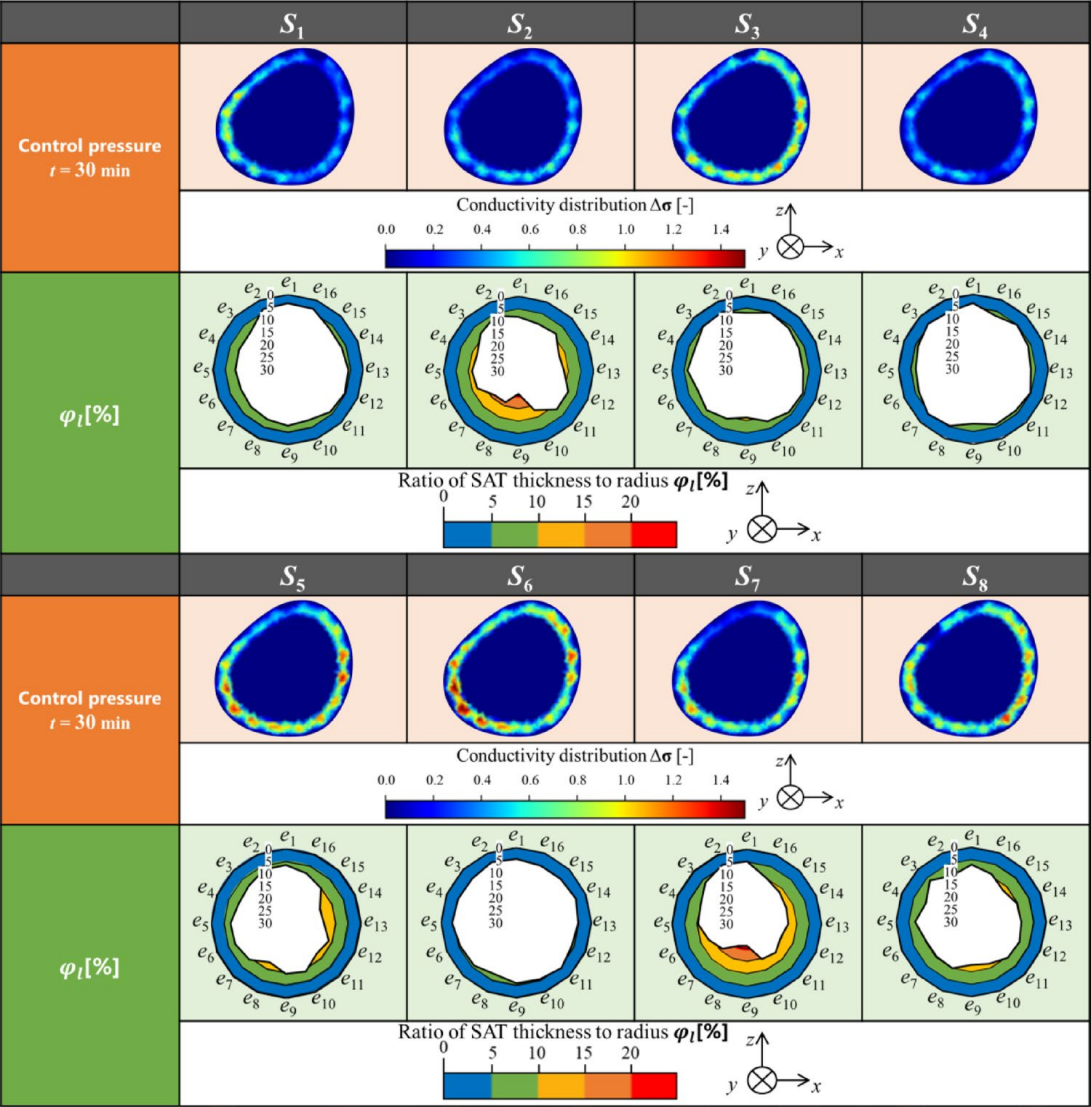
The conductivity distribution $\Delta{\boldsymbol{\sigma}}$ at t_7_ = 30 min as representatives of physiological swelling and the ratio of SAT thickness to radius, φ [%]

Various susceptibilities to physiological swelling are associated with subcutaneous adipose tissue (SAT) thickness because excessive extracellular fluid accumulates mainly above the fascia for physiological swelling-related diseases such as lymphedema [24], in which the impact on the subfascial tissues is minimal [23]. Moreover, the treatment effect of various compression pressures varies depending on subject internal factors, even at the same compression pressure, such as bone position near SAT and viscoelasticity of interstitial fluid in SAT [25]. Therefore, the conductivity distribution $\Delta {\boldsymbol{\upsigma}}$ in SAT during prolonged standing is investigated. In this study, the SBL-EIT image reconstruction utilizes a FEM mesh based on a typical MRI axial-plane image of human calf cross-section [23]; hence, the actual value of SAT thickness should be considered for the interpretation of various susceptibilities to physiological swelling. Figure 8 shows the $\Delta {\boldsymbol{\upsigma}}$ at *t* = 30 min in the case of stocking with control pressure as representatives of physiological swelling and the ratio of SAT thickness to the radius, *φ*_*l*_ [%], which is calculated as follows

9 $${\varphi }_{l}=\frac{{d}_{l}}{r}*100$$

where radius *r* is calculated from the actual circumference measured by a tape measure (Bunkeido Co., Ltd.), assuming the calf cross-section to be a perfect circle. Moreover, SAT thickness ${d}_{l}$ is measured by ultrasound (LOGIQ e Premium, GE Healthcare, Japan) at the cross-section at 16 positions, which is the same as EIT electrode positions.

Comparing SBL-EIT and SAT thickness for each subject, high Δ ${\boldsymbol{\upsigma}}$ is seen mainly in areas with thicker SAT, whereas,$\Delta {\boldsymbol{\upsigma}}$ is low in the anterior calf near the tibia with thinner SAT thickness across all subjects. As for intra-subject comparison, the highest increase in Δ**σ** is observed for each subject at the following positions where SAT is the thickest – lateral calf (electrode *e*_3_ – *e*_8_) for subject *S*_1_ and S_6_, posterior calf (electrode *e*_9_) for subject *S*_2_, posterior calf (electrode *e*_5_ – *e*_13_) for subject *S*_3_, *S*_5_, and *S*_7_. The results are reasonable considering the excessive extracellular fluid accumulates in SAT. Meanwhile, as for intra-subject comparison, the thickest SAT does not necessarily lead to the highest increase in Δ ${\boldsymbol{\upsigma}}$. For example, the thickest SAT is observed for subject *S*_2_, whose increase in Δ ${\boldsymbol{\upsigma}}$ is relatively small among subjects. Conversely, a relatively thinner SAT is observed for subject *S*_6_, whose increase in Δ ${\boldsymbol{\upsigma}}$ is the highest among subjects. Given the above discussion, the result suggests that SAT thickness is certainly critical for susceptibilities to physiological swelling, although other subject internal factors must be considered. Indeed, hydrostatic pressure in the calf is increased during prolonged standing, followed by an increase in the capillary filtration into extracellular fluid [26]. Furthermore, reabsorption in the extracellular fluid is decreased [27] due to the increased osmotic pressure of the extracellular fluid. From the viewpoint of image reconstruction, normalization of the sensitivity matrix [28] and optimization of the measurement pattern [29] would help understand the relationship between the internal muscles, deep veins, etc., and the fat layer and superficial veins. Therefore, other subject internal factors are investigated in future studies.

### Different interpretation of EIT vs BIA and study limitation

Both EIT and BIA utilize a four-electrode measurement method, i.e., tetrapolar technique in BIA, where a small electrical current is applied and the resulting voltage is measured. However, low or negative correlations (Strong—*S*_3_: *R* = − 0.109, *S*_4_: *R* = 0.045, *S*_7_: *R* = − 0.652, *S*_8_: *R* = -0.267, Weak—*S*_7_: *R* = 0.075) between ${\langle {\boldsymbol{\upsigma}}\rangle }^{{\alpha }_{2}}$ and $1/{z}^{\mathrm{BIA}}$ were unexpectedly observed in stockings with strong or weak pressure as shown in Fig. 4c, which can be attributed to the differences in measurement principles between EIT and BIA. BIA considers the body as a cylinder to calculate the impedance ${z}^{\mathrm{BIA}}$ of the entire right leg. Therefore, comparing only the cross-section measured by EIT to the whole of right leg measured by BIA may result in different trends in physiological swelling. For example, while ${\langle {\boldsymbol{\upsigma}}\rangle }^{{\alpha }_{2}}$ is increased in the case of the stocking with strong pressure for *S*_8_, which indicates a tendency towards edema progression, $1/{z}^{\mathrm{BIA}}$ is decreased, which indicates a tendency towards edema reduction as shown in Fig. 4a and b. Therefore, the potential of EIT for more accurate localized evaluation compared to BIA is suggested. Furthermore, the differences in electrodes between EIT and BIA also contribute to some low correlations. While gel electrodes are used in EIT to enhance conductivity, dry electrodes are used in BIA. As a result, the impact of contact impedance is more significant in BIA, leading to potential disturbances in the measurement data and affecting the correlation between ${\langle {\boldsymbol{\upsigma}}\rangle }^{{\alpha }_{2}}$ and $1/{z}^{\mathrm{BIA}}$. In contrast, the gel electrodes used in EIT are likely superior in reducing noise from the subject's skin, thereby providing more reliable measurements.

Even after excluding these outliers, statistically significant differences were still obtained, reinforcing the validity of the results. This suggests that EIT is a reliable method for localized evaluating, while also suggesting that BIA measurements may be influenced by dry electrodes.

Some limitations of this study need to be mentioned. The main limitation is the small number of subjects, including only four males and four females. The experiments in this study are mainly designed to investigate the performance of SBL-EIT for imaging physiological swelling under various stocking compressions. Even though the sample size is small, the correlation between the ⟨**σ**⟩^α2^ and z^BIA^ is moderated to strong; thus, it would be ideal to repeat this study with larger groups. Furthermore, the differences in sex and gender as well as occupational characteristics will be investigated in future studies because those factors affect leg edema during prolonged standing [27]. Also, no relationship was found between Pnet and parameters related to SAT thickness across subjects. Ideally, the thickness of SAT in the calf under stocking compression should have been measured and compared with EIT images. However, due to limitations in the experimental setup, this comparison was not feasible. Future studies should focus on increasing the sample size and incorporating numerical simulation of lower leg model to gain a deeper understanding of the relationship between stocking compression, SAT thickness, and its potential impact on physiological swelling. Additionally, the relationship between SAT thickness under compression and baseline thickness remains unclear. SAT thickness is simply decreased by compression, however, its mechanism is rather complicated because of the poroviscoelastic structure of SAT [25], which will be investigated in a future study. Finally, the limitation of stocking shape is mentioned because the stockings used in this study only cover the ankle. Since the entire calf under the stockings is simultaneously pressurized, the pressure is distributed centripetally and centrifugally, which possibly suggests “toothpaste tube effect” that extracellular fluid is compressed toward both ends of the stocking [28]. The upcoming study involves using stockings that cover the entire leg and conducting multiple cross-sectional EIT measurements instead of a single measurement.

## Conclusions

The conductivity change in subcutaneous adipose tissue (SAT), mainly associated with a change in physiological swelling of the calf, was successfully imaged by the sparse Bayesian learning implemented into electrical impedance tomography (SBL-EIT) during the prolonged standing under the stocking compression. The key findings are as follows:

1. SBL-EIT was applied to physiological swelling imaging under various stocking compressions, considering the differences in skin condition. The spatial-mean conductivity ⟨${\boldsymbol{\upsigma}}$⟩^*α2*^ was highest in the case of stocking with control pressure, followed by weak and strong across all subjects.
2. The ⟨${\boldsymbol{\upsigma}}$⟩^*α2*^ had a strong positive correlation with the conventional inversed impedance 1/*z*^BIA^ by bioelectrical impedance analysis (BIA) (a correlation coefficient 0.528 < *R* < 0.990; *n* = 19 and *p* < 0.05), which was mainly increased during the prolonged standing.
